# Do *Akkermansia* mutants underlie the global metabolic disease epidemic?

**DOI:** 10.1080/19490976.2025.2612582

**Published:** 2026-01-07

**Authors:** Heenam Stanley Kim

**Affiliations:** a Division of Biosystems & Biomedical Sciences, College of Health Sciences, Seoul, Korea

**Keywords:** *Akkermansia* mutant, metabolic disease, gut microbiome, antibiotic-induced damage, purine biosynthesis mutation

## Abstract

Antibiotic-induced mutations in *Akkermansia muciniphila* promote bacterial survival while compromising beneficial host interactions, revealing a potential new link between antibiotic-driven microbiome disruption and metabolic disease. The widespread presence of these mutants suggests that they may contribute to the increasing prevalence of metabolic disorders. If validated in diverse global human cohort studies, these mutants could serve as biomarkers of disease susceptibility and as targets for therapeutic intervention.

The global rise in chronic diseases in recent decades has been linked to alterations in the gut microbiome.^1^ Industrialization and urbanization are believed to have profoundly and cumulatively reshaped the human microbiota across generations through factors such as dietary shifts—especially reduced fiber intake—enhanced hygiene practices, increased rates of Cesarean delivery, and widespread antibiotic use.^1^^,^^2^ Although antibiotic-induced disruption is recognized as a major contributing factor,^1^^,^^3^^,^^4^ the precise nature of this damage and its connection to chronic disease remains poorly understood. Several fundamental questions persist: How does antibiotic-induced damage linger for years? What governs microbiome recovery after disruption? And, in a related context, how does low-dose antibiotic use in livestock promote weight gain? These unresolved issues suggest that taxonomic shifts alone cannot explain the full extent of antibiotic damage, underscoring the need to investigate physiological changes within the gut microbiota, particularly in key bacterial species that exert disproportionate effects on host health.

A recent study by Han et al. provides important insights. They demonstrated that *Akkermansia muciniphila*, a core member of the human gut microbiota, can acquire survival-enhancing mutations in response to antibiotic exposure—while simultaneously losing intrinsic host-beneficial functions.^5^ In a mouse model of metabolic conditions, wild-type *Akkermansia* strains—including the reference strain BAA-835 (MucT) and strain HMI that can be classified as *Akkermansia massiliensis* under a new classification system)—protected against diet-induced metabolic abnormalities, whereas mutant strains did not.^5^ This finding reveals a missing link between antibiotic-induced microbiota alterations and host health, with potential relevance to humans given the widespread and repeated use of antibiotics since World War II (Figure 1).

**Figure 1.**
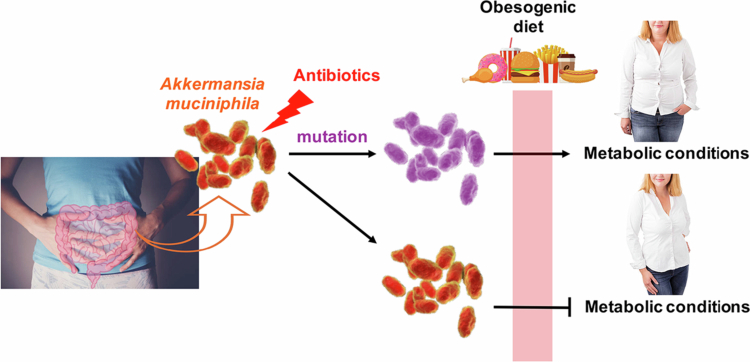
Model illustrating the impact of mutant versus wild-type *Akkermansia muciniphila* strains on host health. Mutant strains selected by antibiotic exposure provide reduced or no protection against an obesogenic diet compared to wild-type strains, as demonstrated in a mouse model by Han et al.^5^

Epidemiological evidence suggests that maternal antibiotic exposure before or during pregnancy, as well as antibiotic exposure in early childhood, can have lasting effects on human health, including an increased risk of early-onset obesity in infants and children.^6‐8^ Such persistence may be explained by the low reversion rates of antibiotic-induced mutations.^9^^,^^10^ Consistent with this view, mutations in *A. muciniphila* appear stable and transmissible across human populations, as reflected by their widespread global prevalence.^5^ Notably, antibiotic exposure is not the only factor causing long-lasting effects; Cesarean section birth has also been associated with an increased risk of chronic diseases such as asthma and obesity later in life.^11^^,^^12^ However, because Cesarean delivery is invariably accompanied by antibiotics, it remains unclear whether the observed effects are due to the delivery mode itself or to the antibiotics. It is therefore highly plausible that these mutants have contributed not only to the long-term consequences of antibiotic-induced damage but also to the global rise in metabolic diseases, including obesity, type II diabetes, and nonalcoholic fatty liver disease. Nonetheless, confirming these hypotheses will require studies across diverse human cohorts worldwide.

Within this context, one factor underlying microbiome recovery after disruption may be the gradual replacement of mutant strains with their wild-type counterparts. Moreover, the results from the mouse model shed light on the long-standing puzzle of how low-dose antibiotics promote weight gain in livestock^13^—by favoring the persistence of functionally impaired strains.

Mechanistically, the primary target of mutation in *A. muciniphila* following exposure to penicillins—the most widely prescribed class of antibiotics worldwide^14^—was the *de novo* purine biosynthesis pathway. Frequent mutations were observed in *purF*, a key gene in this pathway.^5^ Notably, PurF is also a target of the stringent response alarmone (*p*)ppGpp, which inhibits its activity and blocks purine synthesis, as observed in *Escherichia coli.*^15^^,^^16^ Given the central role of the stringent response in bacterial antibiotic tolerance,^16^ its activation through purine synthesis inhibition may represent a molecular mechanism underlying acquired antibiotic insusceptibility in *A. muciniphila*. However, the mechanisms underlying the concomitant loss of host-beneficial functions remain unclear and are likely multifactorial, involving both broader microbiota interactions and host responses. In particular, potential effects on gut barrier function or glucagon-like peptide‑1 (GLP‑1) secretion—which are normally positively regulated by *A. muciniphila* and contribute to improved metabolic health^17^—warrant careful investigation.

The risks associated with antibiotic exposure extend beyond metabolic disorders.^6^ Epidemiological studies link antibiotic use to heightened susceptibility to allergic, autoimmune, and psychiatric conditions, as well as colon cancer—all associated with microbiota disruption and impaired intestinal barrier function.^6^ Intriguingly, *A. muciniphila* has been implicated in protecting against many of these same conditions.^18^ This suggests that mutant *A. muciniphila* strains may also influence disease outcomes beyond metabolic disorders, highlighting their broader relevance for human health.

This emerging mechanism—where antibiotics influence host health by selecting for functionally impaired mutants of essential gut microbes such as *A. muciniphila*—has far-reaching implications for understanding chronic disease susceptibility. If validated in human populations, these findings could open new avenues for diagnostic and therapeutic strategies against metabolic conditions and related disorders, particularly in the context of personalized medicine. Beyond *A. muciniphila*, future research should determine whether similar antibiotic-driven mutations in other keystone microbes, such as *Faecalibacterium prausnitzii* and *Bacteroides thetaiotaomicron*, likewise impair beneficial functions and influence human health.

Ultimately, this study demonstrates that non-taxonomic changes in the microbiota—namely, functional mutations—can exert substantial effects on host physiology. A deeper understanding of the so-called ‘altered gut microbiome’ will bring us closer to unraveling the mechanisms of chronic disease and, ultimately, to developing more effective therapeutic interventions.

## Data Availability

Not applicable.
